# Effect of hyperuricemia on paroxysmal atrial fibrillation after catheter ablation and influence of alcohol consumption

**DOI:** 10.1002/joa3.13092

**Published:** 2024-06-19

**Authors:** Kazuki Shimojo, Itsuro Morishima, Yasuhiro Morita, Yasunori Kanzaki, Hiroyuki Miyazawa, Naoki Watanabe, Naoki Yoshioka, Naoki Shibata, Yoshihito Arao, Ryota Yamauchi, Takuma Ohi, Hiroki Goto, Hoshito Karasawa, Kenji Okumura

**Affiliations:** ^1^ Department of Cardiology Ogaki Municipal Hospital Ogaki Japan; ^2^ Department of Cardiology Tohno Kosei Hospital Mizunami Japan

**Keywords:** alcohol consumption, atrial fibrillation, catheter ablation, hyperuricemia, uric acid

## Abstract

**Background:**

Evidence regarding the association between hyperuricemia and arrhythmia recurrence after catheter ablation for paroxysmal atrial fibrillation (AF) is scarce. We investigated whether hyperuricemia predicts arrhythmia recurrence after catheter ablation for paroxysmal AF and the relationship between hyperuricemia and alcohol consumption in AF recurrence.

**Methods:**

Patients who underwent catheter ablation for paroxysmal AF were divided into the hyperuricemia (index serum uric acid [UA] >7.0 mg/dL; *n* = 114) and control (UA ≤7.0 mg/dL; *n* = 609) groups and were followed for a median of 24 (12–48) months after ablation.

**Results:**

The hyperuricemia group had more patients with an alcohol intake of ≥20 g/day (33.3% vs. 22.7%, *p* = .017) and a lower incidence of AF‐free survival (*p* = .019). Similarly, those with an alcohol intake of ≥20 g/day had a lower incidence of AF‐free survival than other patients. Multivariate Cox regression analysis revealed the following independent predictors of AF recurrence (adjusted hazard ratio, 95% confidence interval): hyperuricemia (1.64, 1.12–2.40), female gender (1.91, 1.36–2.67), brain natriuretic peptide level >100 pg/mL (1.59, 1.14–2.22), and alcohol consumption ≥20 g/day (1.49, 1.03–2.15) (all *p* < .05). In addition, causal mediation analysis revealed that alcohol consumption of ≥20 g/day directly affected AF recurrence, independent of hyperuricemia.

**Conclusions:**

Patients with hyperuricemia may be at a high risk of arrhythmia recurrence after catheter ablation for paroxysmal AF. Although high alcohol consumption may contribute to increased UA levels, the presence of hyperuricemia may independently predict AF recurrence.

## Graphical abstract

Patients with hyperuricemia show increased risk of arrhythmia recurrence after catheter ablation for paroxysmal atrial fibrillation. Although high alcohol consumption (≥20 g/day) may weakly contribute to increased serum uric acid levels, hyperuricemia has independent predictive value.

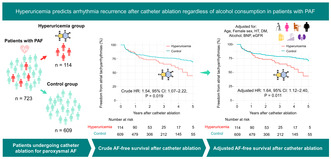

## 1 INTRODUCTION

Atrial fibrillation (AF) is a common cardiac rhythm disturbance that contributes to increased morbidity and mortality. The prevalence of AF is growing and becoming an increasingly serious public health issue worldwide. Catheter ablation is a well‐established therapy for rhythm control in AF, mainly in patients who are refractory or intolerant to antiarrhythmic drugs.^1^, ^2^ Hyperuricemia is defined as a serum uric acid level of >7.0 mg/dL; it affects 20% of men and 5% of women in the Asian population, as well as 20% of men and women in the Western population.^3^, ^4^, ^5^ It can be caused by genetic mutations or polymorphisms that accelerate the production of uric acid or reduce its excretion.^3^, ^6^ Although several studies have revealed an association between increased serum uric acid levels and cardiovascular diseases (including hypertension, coronary artery disease, heart failure, and stroke),^7^ the concept of hyperuricemia as a risk factor for cardiovascular diseases has often been ignored until recently. Hyperuricemia has been shown to be independently associated with new‐onset AF^8^, ^9^; therefore, increased serum uric acid levels are an important independent predictor of AF. Increased oxidative stress has been proposed to play a significant role in AF development.^10^ Serum uric acid is the end product of purine metabolism catalyzed by xanthine oxidase, which plays a central role in uric acid formation and generates superoxides. However, only a few reports regarding the impact of serum uric acid levels on AF recurrence after catheter ablation are available, and the results presented are inconsistent.^11^, ^12^, ^13^, ^14^, ^15^ Moreover, alcohol consumption has recently emerged as a possible predictor of AF recurrence after catheter ablation as a result of multifactorial effects.^16^, ^17^, ^18^ Generally, hyperuricemia is related to alcohol consumption,^19^ although evidence regarding the relationship between hyperuricemia and alcohol consumption in patients with AF recurrence after catheter ablation remains lacking. In this study, we aimed to clarify the contribution of hyperuricemia to the recurrence of arrhythmia after catheter ablation for paroxysmal AF. Furthermore, we performed a causal mediation analysis to identify the mediating effects of alcohol consumption on hyperuricemia in terms of AF recurrence.^20^

## 2 METHODS

This retrospective study included 723 consecutive patients who underwent index radiofrequency or cryoballoon ablation for paroxysmal AF at the Ogaki Municipal Hospital in Japan between November 2013 and July 2020. Paroxysmal AF was defined as episodes of AF lasting for <7 days with a subsequent reversion to sinus rhythm.^2^ Data were retrieved from the Ogaki Catheter Ablation Database. Data on the baseline clinical characteristics of the patients were collected by interviewing the patients and investigating their medical history within 1 week before hospitalization for catheter ablation procedures. Daily alcohol consumption was calculated using data collected from the patients through questionnaires. All parameters were measured using standard laboratory methods. We set 20 g/day as the cut‐off point for alcohol intake because the Japanese Ministry of Health, Labour and Welfare defines an alcohol intake of <20 g/day as modest alcohol consumption.^21^ On the basis of their index serum uric acid level, the patients were divided into the hyperuricemia group (uric acid >7.0 mg/dL) and the control group (uric acid ≤7.0 mg/dL).^22^

After written informed consent was obtained from the patients, ablation procedures were performed under local anesthesia with mild conscious sedation. Most patients underwent circumferential pulmonary vein isolation with point‐by‐point applications performed using an irrigation tip catheter to create contiguous lesions under the guidance of a three‐dimensional mapping system (CARTO System, Biosense Webster, Diamond Bar, CA, USA, or NavX System, St. Jude Medical, Saint Paul, MN, USA). The remaining patients underwent individual pulmonary vein isolation using a second‐generation cryoballoon ablation catheter (Arctic Front Advance: Medtronic, Minneapolis, MN, USA). Additional procedures (e.g., posterior wall isolation, left atrial roof or anterior linear ablation, superior vena cava isolation, nonpulmonary vein foci ablation, or cavotricuspid isthmus ablation) were performed at the operators' discretion.

Patients underwent periodic follow‐up at the outpatient clinic at 1, 3, 6, 12, 24, 36, 48, and 60 months after the ablation procedure in accordance with our standard protocol.^23^ Discontinuation of antiarrhythmic drugs, if prescribed, was strongly encouraged within 3 months after the procedure. If the patients presented with symptoms, ambulatory electrocardiographic monitoring was performed using a portable electrocardiography device (HCG‐801; Omron Healthcare, Kyoto, Japan) to correlate the findings with the symptoms. Both 12‐lead electrocardiogram and 24‐h Holter monitoring were performed at each visit. The primary endpoint of the study was AF recurrence, which was defined as any documented episode of atrial tachyarrhythmia lasting for ≥30 s with or without the use of antiarrhythmic drugs, with a 90‐day blanking period following the first ablation. All patients were followed up for a minimum of 6 months and a maximum of 5 years. The study protocol complied with the Declaration of Helsinki and was approved by the Institutional Review Board of the Ogaki Municipal Hospital.

Continuous variables are expressed as mean ± standard deviation or as median and interquartile range. Categorical variables are expressed as counts and percentages. Continuous variables were compared using the Student's *t*‐test for parametric data and the Mann–Whitney *U* test for nonparametric data. The chi‐square or Fisher's exact test was used for categorical data. The Kaplan–Meier curve was truncated at 60 months with a 3‐month blanking period, and log‐rank significance testing was performed. Univariate and multivariate logistic regression analyses were performed to assess the associations between hyperuricemia and the baseline variables. For AF recurrence, univariate and multivariate regression analyses were performed using Cox proportional hazards modeling. The model for multivariate analysis, including age and gender, was based on the Akaike information criterion. We adjusted the survival curves to improve our understanding of the multivariate Cox model results, including the remaining multivariate variables after backward stepwise feature selection.^24^

To explore whether the effect of alcohol consumption on AF recurrence was mediated by hyperuricemia, we performed a causal mediation analysis to further characterize the causality relationship. A causal mediation analysis is a method of differentiating the total effect of a treatment into direct and indirect effects.^20^ The indirect effect on the outcome is mediated via a mediator. This type of analysis is used to identify and explain the mechanism underlying an observed relationship between an independent variable and a dependent variable via the inclusion of another hypothetical variable. This analysis estimates the average causal mediation effect, average direct effect, and total effect.

All statistical analyses were performed using EZR (Saitama Medical Center, Jichi Medical University, Saitama, Japan), a graphical user interface for R (R Foundation for Statistical Computing, Vienna, Austria), which is a modified version of the R commander designed to add statistical functions frequently used in biostatistics. All *p*‐values were two‐sided, and the significance level was set at *p* < .05.

## 3 RESULTS

The baseline characteristics of the patients in both groups are compared in Table 1. The proportions of men and those with an alcohol intake of ≥20 g/day were higher in the hyperuricemia group than in the control group. The renal function was worse in the hyperuricemia group than in the control group, as evidenced by the increased creatinine level and decreased estimated glomerular filtration rate in the hyperuricemia group. Cardiac dysfunction was also more advanced in the hyperuricemia group than in the control group, as evidenced by the increased left atrial diameter, left atrial volume, and brain natriuretic peptide level; decreased left ventricular ejection fraction; and higher incidence of a history of heart failure in the hyperuricemia group. Several medications used by the patients, such as angiotensin‐converting enzyme inhibitors, angiotensin receptor II blockers, diuretics, and class III antiarrhythmic agents, were associated with hyperuricemia.

**TABLE 1:** Patient characteristics before the catheter ablation procedure.

Parameters	All (*n* = 723)	Hyperuricemia group (*n* = 114)	Control group (*n* = 609)	*p*‐value
*Patient characteristics*				
Age (years)	68.1 ± 10.9	67.3 ± 12.6	68.3 ± 10.5	.35
Female gender, *n* (%)	244 (33.7)	24 (21.1)	220 (36.1)	.002
BMI (kg/m^2^)	23.9 ± 3.5	24.6 ± 3.8	23.8 ± 3.5	.041
CHA_2_DS_2_‐VASc score	2.5 ± 1.6	2.4 ± 1.7	2.5 ± 1.6	.89
Hypertension, *n* (%)	430 (59.5)	74 (64.9)	356 (58.5)	.21
Diabetes mellitus, *n* (%)	122 (16.9)	17 (14.9)	105 (17.2)	.59
History of heart failure, *n* (%)	99 (13.7)	32 (28.1)	67 (11.0)	.001
Systemic embolism or stroke/TIA, *n* (%)	77 (10.7)	11 (9.6)	66 (10.8)	.87
Alcohol ≥20 g/day, *n* (%)	176 (24.3)	38 (33.3)	138 (22.7)	.017
BNP (pg/mL)	47.6 [20.7–103.4]	62.2 [23.3–160.5]	46.2 [20.5–92.4]	.017
UA (mg/dL)	5.6 ± 1.5	7.9 ± 0.9	5.2 ± 1.1	.001
UN (mg/dL)	15.8 [12.6–19.2]	17.6 [13.9–21.7]	15.5 [12.7–18.8]	.001
Creatinine (mg/dL)	0.83 [0.71–0.98]	0.98 [0.85–1.12]	0.80 [0.69–0.94]	.001
eGFR (mL/min/1.73 m^2^)	65.0 ± 18.7	57.2 ± 19.1	66.5 ± 18.3	.001
Albumin (g/dL)	4.3 ± 0.3	4.3 ± 0.4	4.3 ± 0.3	.37
CRP (mg/dL)	0.06 [0.03–0.14]	0.08 [0.05–0.14]	0.06 [0.03–0.14]	.018
LAD (mm)	39.0 [34.9–43.3]	40.7 [35.7–45.0]	38.7 [34.7–43.0]	.011
LAV (mL)	91.0 [77.0–109.0]	101.3 [82.1–130.6]	95.2 [79.7–115.3]	.019
LVEF (%)	63.1 ± 9.3	61.8 ± 8.8	64.0 ± 7.0	.004
*Medications*				
Ca blockers, *n* (%)	318 (44.0)	55 (48.2)	263 (43.2)	.36
ACE‐I/ARB, *n* (%)	315 (43.6)	69 (60.5)	246 (40.4)	.001
β‐blockers, *n* (%)	262 (36.2)	56 (49.1)	206 (33.9)	.003
Diuretics, *n* (%)	121 (16.7)	36 (31.6)	85 (14.0)	.001
Loop diuretics, *n* (%)	74 (10.2)	27 (23.7)	47 (7.7)	.001
Thiazides, *n* (%)	44 (6.1)	12 (10.5)	32 (5.3)	.051
MRA, *n* (%)	51 (7.1)	15 (13.2)	36 (5.9)	.009
Tolvaptan, *n* (%)	11 (1.5)	5 (4.4)	6 (1.0)	.019
SGLT2‐I, *n* (%)	2 (0.3)	1 (0.9)	1 (0.2)	.29
Urate‐lowering drugs, *n* (%)	80 (11.1)	14 (12.4)	66 (10.9)	.63
Antiarrhythmic drugs, *n* (%)				
Class I	147 (20.3)	23 (20.2)	124 (20.4)	.99
Class III	16 (2.2)	7 (6.1)	9 (1.5)	.007

Abbreviations: ACE‐I, angiotensin‐converting enzyme inhibitor; ARB, angiotensin receptor II blocker; BMI, body mass index; BNP, brain natriuretic peptide; Ca, calcium; CRP, C reactive protein; eGFR, estimated glomerular filtration rate; LAD, left atrial diameter; LAV, left atrial volume; LVEF, left ventricular ejection fraction; MRA, mineralocorticoid receptor antagonist; SGLT2‐I, sodium–glucose cotransporter 2 inhibitor; TIA, transient ischemic attack; UA, uric acid; UN, urea nitrogen.

The independent predictors for hyperuricemia were determined using univariate and multivariate logistic regression analyses (Table 2) and included female gender (adjusted odds ratio: 0.37, 95% confidence interval: 0.22–0.65, *p* < .001), history of heart failure (adjusted odds ratio: 2.43, 95% confidence interval: 1.33–4.45, *p* = .004), reduced estimated glomerular filtration rate (adjusted odds ratio: 2.86, 95% confidence interval: 1.78–4.60, *p* < .001), diuretic prescription (adjusted odds ratio: 1.91, 95% confidence interval: 1.08–3.40, *p* = .027), and urate‐lowering drug use (adjusted odds ratio: 0.44, 95% confidence interval: 0.22–0.88, *p* = .020). Alcohol consumption was a positive significant predictor of hyperuricemia in the univariate model; while it remained in the final multivariate model, its contribution was not significant.

**TABLE 2:** Univariate and multivariate predictors of hyperuricemia in patients undergoing catheter ablation for paroxysmal atrial fibrillation.

Parameters	Univariate		Multivariate	
	OR (95% CI)	*p*‐value	OR (95% CI)	*p*‐value
Age ≥75 years	1.02 (0.66–1.57)	.95	0.66 (0.40–1.09)	.11
Female gender	0.47 (0.29–0.76)	.002	0.37 (0.22–0.65)	.001
BMI ≥25 kg/m^2^	1.17 (0.77–1.77)	.46		
Hypertension	1.31 (0.87–2.00)	.20		
Diabetes	0.84 (0.48–1.47)	.54		
History of heart failure	3.16 (1.95–5.11)	.001	2.43 (1.33–4.45)	.004
Alcohol intake ≥20 g/day	1.71 (1.11–2.63)	.016	1.44 (0.89–2.34)	.14
BNP ≥100 pg/mL	1.96 (1.28–2.98)	.002		
eGFR <60 mL/min/m^2^	2.47 (1.65–3.71)	.001	2.86 (1.78–4.60)	.001
CRP >0.3 mg/dL	1.93 (1.18–3.17)	.009	1.64 (0.98–2.74)	.06
LAD >40 mm	1.58 (1.06–2.37)	.025	1.43 (0.93–2.20)	.11
LVEF <50%	2.48 (1.25–4.92)	.009		
Diuretics	2.85 (1.80–4.49)	.001	1.91 (1.08–3.40)	.027
Urate‐lowering drugs	1.16 (0.63–2.14)	.64	0.44 (0.22–0.88)	.020

Abbreviations: BMI, body mass index; BNP, brain natriuretic peptide; CI, confidence interval; CRP, C reactive protein; eGFR, estimated glomerular filtration rate; LAD, left atrial diameter; LVEF, left ventricular ejection fraction; OR, odds ratio.

The procedural characteristics of catheter ablation did not differ significantly according to the presence or absence of hyperuricemia (Table 3). Pulmonary vein isolation was successfully performed in all patients. Contact force‐guided pulmonary vein isolation and the cavotricuspid isthmus line tended to be more prevalent in the hyperuricemia group than in the control group, but this difference was not significant. At the end of the 90‐day blanking period, antiarrhythmic drugs were prescribed to seven patients (6.1%) in the hyperuricemia group and three patients (0.5%) in the control group (*p* < .01).

**TABLE 3:** Comparison of catheter ablation procedures between patients with and without hyperuricemia.

Procedures	All (*n* = 723)	Hyperuricemia group (*n* = 114)	Control group (*n* = 609)	*p*‐value
PV isolation, *n* (%)	723 (100)	114 (100)	609 (100)	.057
Contact force‐guided PV isolation, *n* (%)	363 (50.2)	69 (60.5)	294 (48.3)	
Noncontact force‐guided PV isolation, *n* (%)	105 (14.5)	14 (12.3)	91 (14.9)	
Cryoballoon ablation, *n* (%)	255 (35.3)	31 (27.2)	224 (36.8)	
Posterior wall isolation, *n* (%)	16 (2.2)	2 (1.8)	14 (2.3)	.99
LA lines, *n* (%)	4 (0.6)	0 (0)	4 (0.7)	.99
Cavotricuspid isthmus line, *n* (%)	509 (70.4)	89 (78.1)	420 (69.0)	.057
SVC isolation, *n* (%)	28 (3.9)	4 (3.5)	24 (3.9)	.99
Non‐PV/SVC foci ablation, *n* (%)	10 (1.4)	0 (0)	10 (1.6)	.38

Abbreviations: LA, left atrial; PV, pulmonary vein; SVC, superior vena cava.

**FIGURE 1:**
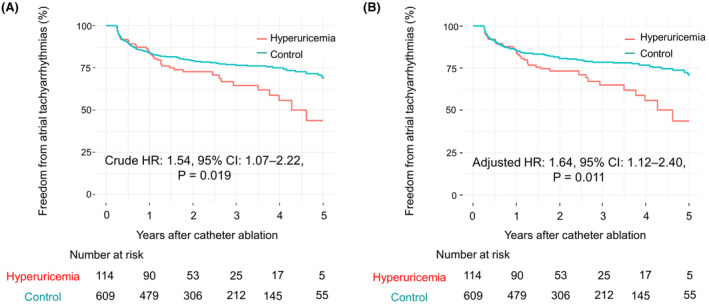
Comparison of Kaplan–Meier curves of cumulative atrial fibrillation‐free survival after catheter ablation between patients with and without hyperuricemia. (A) Unadjusted model. Atrial fibrillation‐free survival was significantly lower in the hyperuricemia group than in the control group. (B) Adjusted model. A multivariate Cox regression model, including age, gender, hypertension, diabetes mellitus, brain natriuretic peptide level, estimated glomerular filtration rate, and alcohol consumption, was developed using backward stepwise feature selection. This model revealed hyperuricemia as an independent predictor of atrial fibrillation recurrence. CI, confidence interval; HR, hazard ratio.

AF recurrence was observed in 37 (32.5%) and 136 (22.3%) patients in the hyperuricemia and control groups, respectively, during a median follow‐up of 2 (interquartile range: 1.0–4.0) years. Figure 1 shows the comparison of the Kaplan–Meier curves of cumulative AF‐free survival between the two groups. The AF‐free survival in the hyperuricemia group was significantly lower than that in the control group (crude hazard ratio: 1.54, 95% confidence interval: 1.07–2.22, *p* = .019; Figure 1A).

**FIGURE 2:**
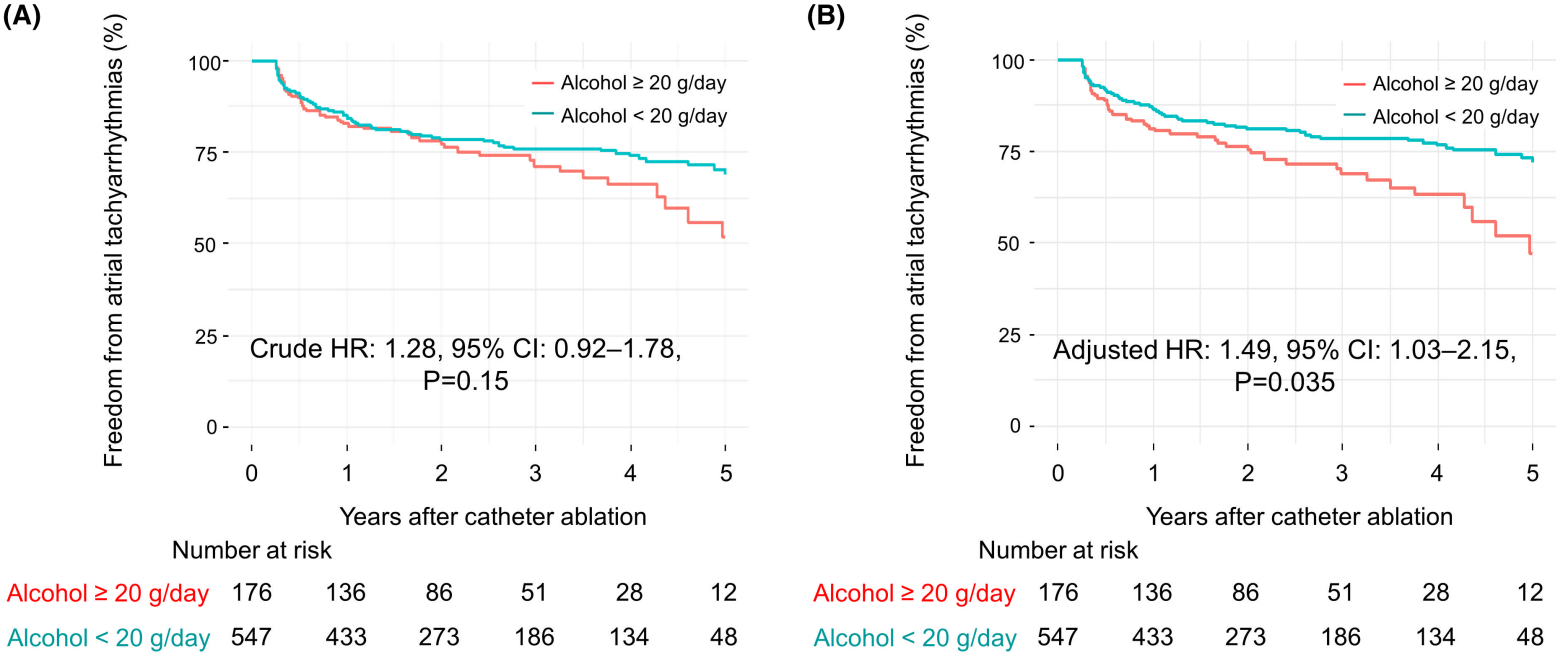
Comparison of Kaplan–Meier curves of cumulative atrial fibrillation‐free survival after catheter ablation between patients with an alcohol intake of ≥20 g/day and <20 g/day. (A) Unadjusted model. Atrial fibrillation‐free survival in the presence of an alcohol intake of ≥20 g/day was not significantly lower than that in the absence of an alcohol intake of ≥20 g/day. (B) Adjusted model. A multivariate Cox regression model, including age, gender, hypertension, diabetes mellitus, brain natriuretic peptide level, estimated glomerular filtration rate, and hyperuricemia, was developed using backward stepwise feature selection. This model revealed that alcohol intake ≥20 g/day was an independent predictor of atrial fibrillation recurrence. CI, confidence interval; HR, hazard ratio.

**FIGURE 3:**
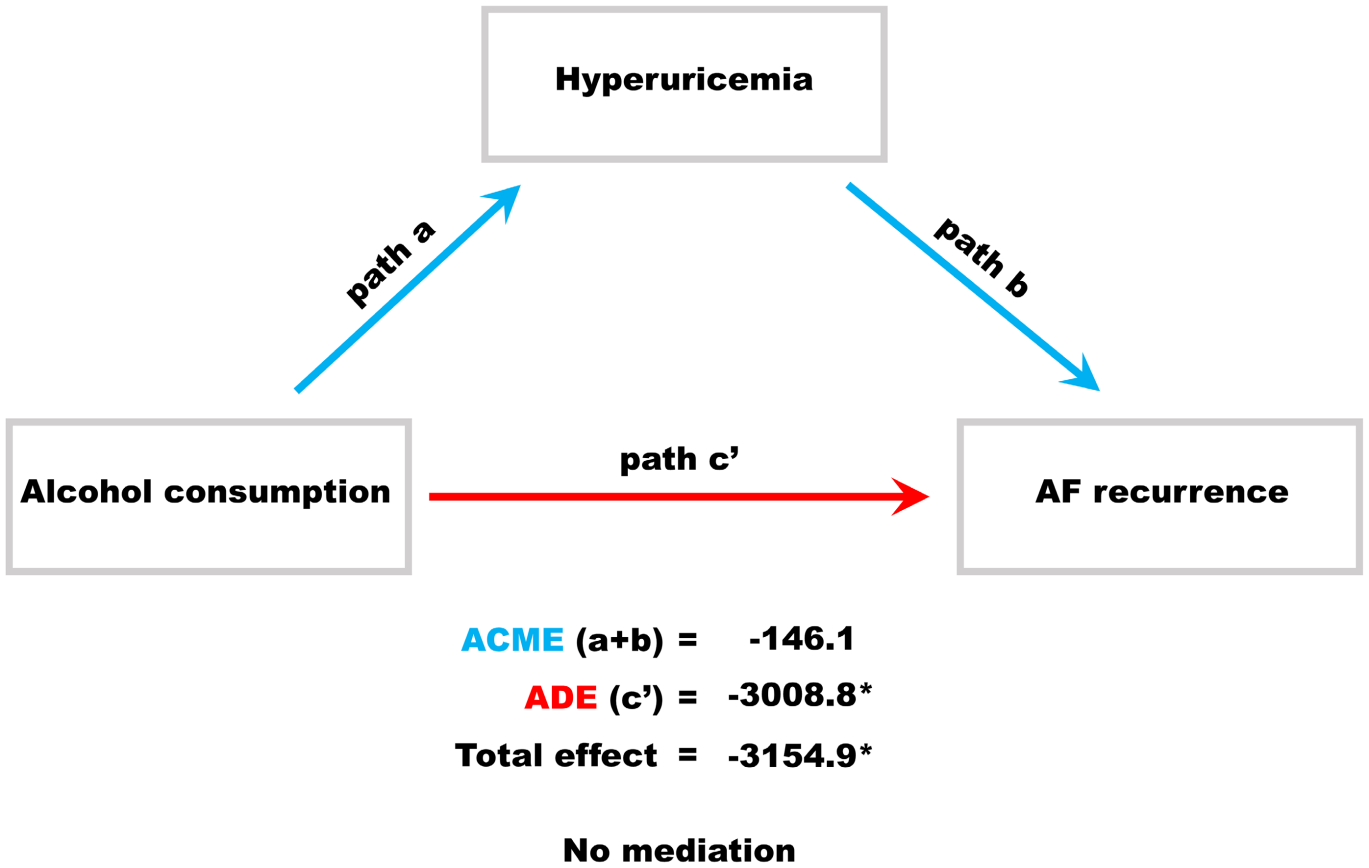
Causal mediation analysis for alcohol consumption, hyperuricemia, and AF recurrence. Causal mediation analysis is a method of differentiating the total effect of treatment into direct and indirect effects. Causal mediation analysis revealed that alcohol consumption (alcohol intake ≥20 g/day) had a direct effect on AF recurrence after the catheter ablation procedure (*p* = .028 for ADE) and did not mediate the effect of hyperuricemia on AF recurrence after the procedure (*p* = .23 for ACME). These results may indicate that hyperuricemia is a predictor of AF recurrence after catheter ablation independent of alcohol consumption. ACME, average causal mediation effect; ADE, average direct effect; AF, atrial fibrillation. *, Significant difference.

The predictors of AF recurrence, as determined by Cox regression analysis, are shown in Table 4. The significant univariate predictors of AF recurrence other than hyperuricemia were female gender, brain natriuretic peptide level >100 pg/mL, history of heart failure, and prescription of diuretics. An alcohol intake of ≥20 g/day was not a significant univariate predictor of AF recurrence (crude hazard ratio: 1.28, 95% confidence interval: 0.92–1.78, *p* = .15; Figure 2A). A multivariate Cox regression model was developed using backward stepwise feature selection with the Akaike information criterion. The independent predictors of AF recurrence were female gender (adjusted hazard ratio: 1.91, 95% confidence interval: 1.36–2.67, *p* < .001), alcohol intake ≥20 g/day (adjusted hazard ratio: 1.49, 95% confidence interval: 1.03–2.15, *p* = .035; Figure 2B), brain natriuretic peptide level >100 pg/mL (adjusted hazard ratio: 1.59, 95% confidence interval: 1.14–2.22, *p* = .006), and hyperuricemia (adjusted hazard ratio: 1.64, 95% confidence interval: 1.12–2.40, *p* = .011; Figure 1B). Causal mediation analysis (Figure 3) showed that an alcohol intake of ≥20 g/day exerted direct effects on AF recurrence after the catheter ablation procedure (*p* = .028 for average direct effect) and did not mediate the effects of hyperuricemia (*p* = .23 for average causal mediation effect).

**TABLE 4:** Predictors of arrhythmia recurrence after catheter ablation for paroxysmal atrial fibrillation.

Parameters	Univariate		Multivariate	
	HR (95% CI)	*p*‐value	HR (95% CI)	*p*‐value
Age ≥75 years	1.13 (0.82–1.56)	.44	1.07 (0.75–1.54)	.70
Female gender	1.58 (1.17–2.13)	.003	1.91 (1.36–2.67)	.001
BMI ≥25 kg/m^2^	0.87 (0.63–1.19)	.38		
Hypertension	0.81 (0.60–1.10)	.18	0.74 (0.54–1.01)	.062
Diabetes	1.22 (0.83–1.78)	.30	1.34 (0.91–1.99)	.14
History of heart failure	1.66 (1.14–2.42)	.008		
Alcohol intake ≥20 g/day	1.28 (0.92–1.78)	.15	1.49 (1.03–2.15)	.035
BNP ≥100 pg/mL	1.73 (1.66–2.35)	.001	1.59 (1.14–2.22)	.006
Hyperuricemia	1.54 (1.07–2.22)	.019	1.64 (1.12–2.40)	.011
eGFR <60 mL/min/m^2^	0.91 (0.67–1.25)	.57	0.75 (0.53–1.07)	.11
CRP >0.3 mg/dL	1.07 (0.77–1.49)	.69		
LAD >40 mm	1.25 (0.93–1.68)	.15		
LVEF <50%	1.39 (0.79–2.44)	.26		
Diuretics	1.44 (1.01–2.05)	.046		
Urate‐lowering drugs	1.00 (0.62–1.61)	1.00		

Abbreviations: BMI, body mass index; BNP, brain natriuretic peptide; CI, confidence interval; CRP, C reactive protein; eGFR, estimated glomerular filtration rate; HR, hazard ratio; LAD, left atrial diameter; LVEF, left ventricular ejection fraction.

**FIGURE 4:**
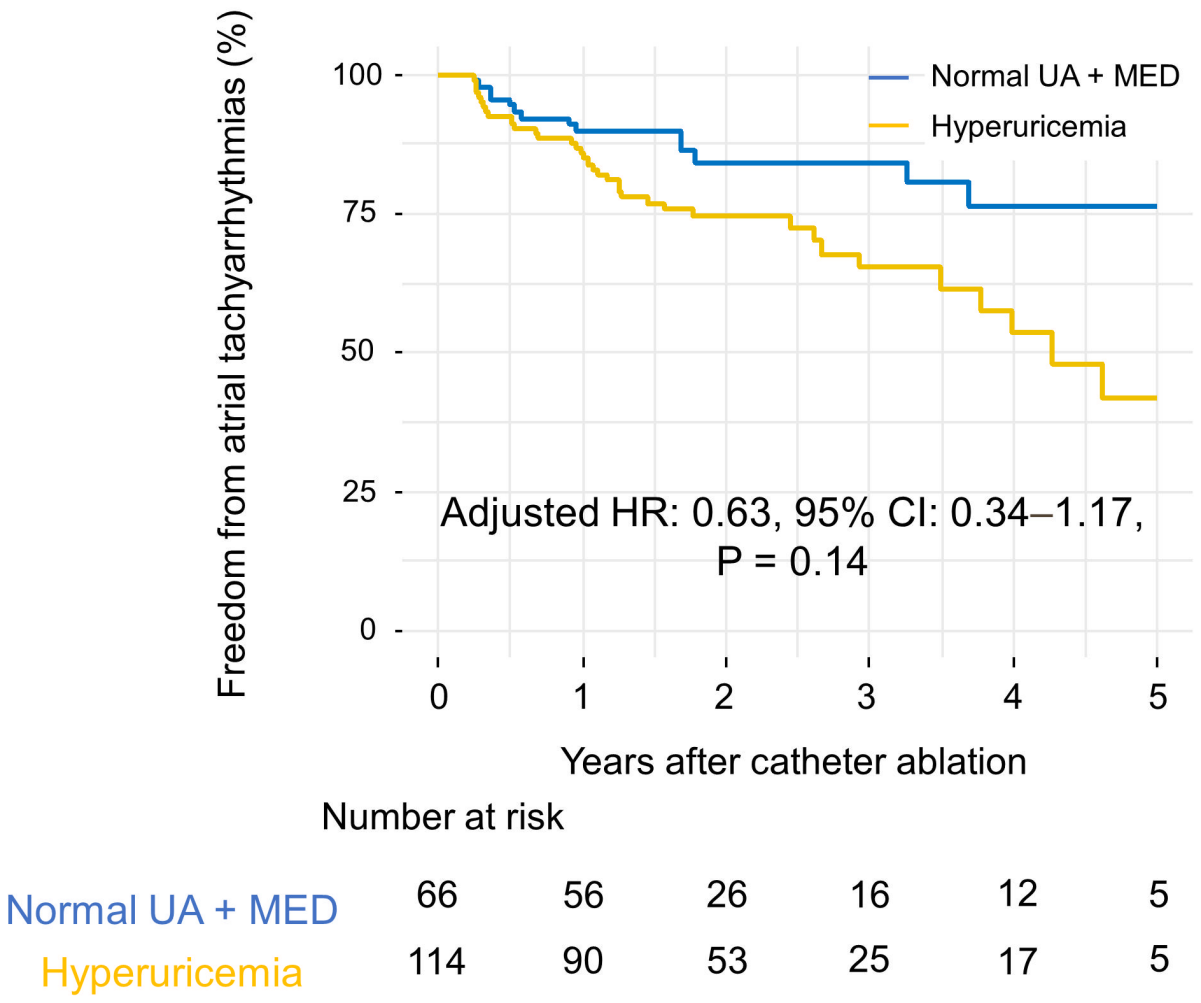
Comparison of Kaplan–Meier curves of cumulative atrial fibrillation‐free survival between patients on urate‐lowering drugs in the control group and those in the hyperuricemia group. Patients on urate‐lowering drugs in the control group, who otherwise should have had hyperuricemia, showed better atrial fibrillation‐free survival than those in the hyperuricemia group; however, this difference was not significant owing to limited data availability. CI, confidence interval; HR, hazard ratio; MED, medication; UA, uric acid.

The control group included 66 patients who were on urate‐lowering drugs. The AF‐free survival of this subgroup was better than that of the hyperuricemia group, although the difference was not significant (adjusted hazard ratio: 0.63, 95% confidence interval: 0.34–1.17, *p* = .14; Figure 4).

## 4 DISCUSSION

This study, which consisted of patients undergoing index catheter ablation for paroxysmal AF, revealed the following important findings. First, pre‐procedural hyperuricemia was strongly associated with male gender, impaired heart and renal function, and diuretic prescription; moreover, it was weakly associated with older age, an enlarged left atrium, and alcohol consumption. Second, hyperuricemia was an independent predictor of arrhythmia recurrence after catheter ablation. Finally, this predictive value was valid irrespective of alcohol consumption, which is another emerging predictor of AF recurrence.

To the best of our knowledge, only four cohort studies have investigated the effect of hyperuricemia on AF recurrence following catheter ablation for AF^11^, ^12^, ^13^, ^14^; these have been summarized in a meta‐analysis by Zhao et al.^15^ Zhao et al. concluded that elevated serum uric acid levels were not associated with AF recurrence. However, three of the four studies affirmed the involvement of high uric acid levels in the development of AF recurrence,^11^, ^12^, ^13^ whereas the remaining study did not find a significant effect of hyperuricemia.^14^ The validity of this meta‐analysis^15^ may be limited by heterogeneity across the studies, such as in the follow‐up periods, ablation techniques, and types of AF (paroxysmal and nonparoxysmal AF). In addition, the participants of these four studies underwent catheter ablation more than a decade ago. With significant developments in catheter ablation techniques, the durability of pulmonary vein isolation has improved over time.^2^, ^25^ Thus, our study has the following strengths over the previous studies: 1) exclusive inclusion of patients with paroxysmal AF, 2) relatively large cohort size, and 3) greater relevance to current developments in ablation procedures.

A dose‐dependent relationship between alcohol intake and incident AF is well recognized.^26^ Recently, alcohol consumption has emerged as a predictor of arrhythmia recurrence after catheter ablation for AF.^16^, ^17^, ^18^ Alcohol abstinence has also been reported to be effective in the secondary prevention of AF with medication^27^ and/or catheter ablation.^17^ The results of the present study are consistent with these findings. Patients with an alcohol consumption of ≥20 g/day were approximately 1.6 times more likely to have AF recurrence than those with an alcohol consumption of <20 g/day. Furthermore, causal mediation analysis revealed that alcohol consumption had a direct effect on AF recurrence after catheter ablation and did not mediate the postablation effects of hyperuricemia. Therefore, the association between hyperuricemia and recurrent AF after catheter ablation was not influenced by alcohol consumption.

The association between hyperuricemia and AF recurrence may be multifactorial. Serum uric acid is the end product of purine degradation catalyzed by xanthine oxidase, which has been reported to be correlated with oxidative stress^28^ and triggers the elevation of systemic inflammatory markers. In addition, elevated uric acid levels could lead to endothelial dysfunction and activation of the renin–angiotensin system.^29^ Conceptually, elevated uric acid levels can contribute to the pathophysiology of inflammatory signaling‐induced changes through inflammation‐independent mechanisms.^30^ Recently, further explanation regarding the association between high uric acid levels and AF has emerged. Intracellular accumulation of uric acid via the activation of urate transporters is posited to cause cell injury through several signaling pathways.^31^ In addition to those in vascular smooth muscle and endothelial cells, urate transporters are also expressed in renal tubular cells.^32^, ^33^ Intracellular uric acid uptake by urate transporters reportedly enhances Kv1.5 protein expression, which may be attributable to the shortening of action potential duration, resulting in the initiation or sustainment of AF.^34^ In this study, the patients on urate‐lowering drugs in the control group, who otherwise should have had hyperuricemia, showed better AF‐free survival than those in the hyperuricemia group; this suggests a direct causal relationship between the serum uric acid level and AF recurrence. However, this difference was not significant owing to limited data availability.

In contrast, the serum uric acid level might be a surrogate marker for the risk of AF recurrence. In this study, we determined the factors affecting the serum uric acid level. The positive significant independent factors were male gender, lower estimated glomerular filtration rate, a history of heart failure, and diuretic medication use; conversely, a significant negative factor was urate‐lowering drug use. Renal failure (reflected by a lower estimated glomerular filtration rate) and a history of heart failure have been identified as factors associated with the development of AF or AF recurrence after catheter ablation.^35^, ^36^ Alcohol consumption also contributed to hyperuricemia, although the correlation was weak. Moreover, it has been shown to increase AF recurrence after catheter ablation.^16^, ^17^, ^18^ Considered together, preablation hyperuricemia might reflect the combined risk of AF recurrence from cardiac and renal dysfunctions, alcohol consumption, and medications, rather than uric acid acting alone to develop AF recurrence.

This observational retrospective study has some limitations. First, the patients included took several medications that could have affected their uric acid levels. In particular, some prescription urate‐lowering drugs are very effective at reducing serum uric acid levels and may have interfered with the assessment of hyperuricemia. Second, we determined the quantity of alcohol consumption using self‐reported data, rather than by objective blood or urine sampling. Possible inaccurate reporting may have caused bias in the analysis. In addition, the diagnosis of AF was only based on findings from 12‐lead electrocardiogram, 24‐h Holter electrocardiogram monitoring per periodical outpatient visits, and ambulatory electrocardiographic monitoring using a portable electrocardiography device in symptomatic patients; accordingly, asymptomatic atrial tachyarrhythmia may have been missed. Therefore, we may have underestimated the recurrence of atrial tachyarrhythmia in this study.

## 5 CONCLUSIONS

Patients with hyperuricemia may be at a higher risk of arrhythmia recurrence after catheter ablation for paroxysmal AF compared with patients with normal uric acid levels. Although high alcohol consumption (≥20 g/day), another predictor of AF recurrence, may weakly contribute to increased serum uric acid levels, the presence of hyperuricemia may be an independent predictor of recurrent arrhythmias. Studies incorporating interventions to lower the serum uric acid level are warranted to further evaluate whether hyperuricemia is a therapeutic target or a simple biomarker of AF recurrence.

## FUNDING INFORMATION

None received.

## CONFLICT OF INTEREST STATEMENT

The authors declare that there are no conflicts of interest.

## ETHICS STATEMENT

The study protocol complied with the Declaration of Helsinki and was approved by the Institutional Review Board of the Ogaki Municipal Hospital (No. 20220825‐10).

## INFORMED CONSENT

All patients provided written informed consent.

## ANIMAL STUDIES

Not applicable.

## Data Availability

The data supporting the findings of this study are available from the corresponding author upon reasonable request.
